# Crosstalk Between Gastric Cancer and Adjacent Mucosa Reveals EDN1‐EDNRA‐Mediated Regulation of Cancer Stemness and Immunomodulation Networks

**DOI:** 10.1111/jcmm.70547

**Published:** 2025-04-17

**Authors:** Xiaobin Zhu, Yating Zhang, Hanlin Liao, Jing Hu, Xiao Xiao

**Affiliations:** ^1^ Department of Spine Surgery and Musculoskeletal Tumor Zhongnan Hospital of Wuhan University Wuhan City Hubei Province People's Republic of China; ^2^ Institute of Health Inspection and Testing Hubei Provincial Center for Disease Control and Prevention Wuhan Hubei People's Republic of China; ^3^ Department of Medical Services Section Taihe Hospital, Hubei University of Medicine Shiyan People's Republic of China; ^4^ Occupational Disease Prevention and Control Department Center for Disease Control and Prevention of Yangtze River Navigation Administration Wuhan Hubei People's Republic of China; ^5^ Department of Laboratory Medicine Zhongnan Hospital of Wuhan University Wuhan People's Republic of China

**Keywords:** apoptosis, cell–cell communication, endothelin receptor a, gastric cancer, immunomodulation, single‐cell RNA sequencing, tumour microenvironment

## Abstract

Within the complex system of gastric cancer, the tumour microenvironment mediates a complex network of cellular interactions, yet its tissue‐specific intracellular communication patterns have remained poorly understood. Leveraging cutting‐edge single‐cell RNA sequencing, we investigated two independent research studies (GSE183904 and GSE184198), creating an unprecedented map of cellular crosstalk across gastric cancer tissues, their adjacent normal tissue and gastric mucosa (GM). Our systematic analysis revealed two distinct patterns: 7557 distinct interactions from normal tissue to tumour cells, while gastric mucosa engaged in 7320 unique interactions with malignant conditions. Within this cellular network, the endothelin pathway emerged as a key regulator, specifically increased in gastric mucosa‐to‐tumour interaction. The Cancer Genome Atlas data demonstrated that patients harbouring elevated EDNRA expression faced significantly poorer outcomes. EDNRA, previously underexplored in this context, showed remarkable upregulation across diverse gastric cancer cell lines. Through experimental validation, we demonstrated that EDNRA overexpression, when stimulated by endothelin‐1, dramatically accelerated the proliferation of human gastric epithelial GSE‐1 cells. Conversely, pharmacological inhibition of EDNRA using ABT‐627 suppressed both NCI‐N87 and MKN‐28 gastric cancer cells proliferation. Further mechanistic investigation revealed the molecular mechanism of ABT‐627: simultaneously triggering both extrinsic and intrinsic apoptotic cascades. TISIDB analysis revealed significant positive correlations between EDNRA and multiple immunostimulators, suggesting the role of EDNRA in immunomodulation networks. These findings reveal a previously unidentified connection between gastric mucosa and tumour progression, positioning EDNRA not only as a molecular target, but also as a critical mediator of tissue‐specific cancer communication. In conclusion, EDNRA functions as both a regulatory factor and therapeutic target, offering a promising therapeutic avenue for gastric cancer intervention.

## 1 Introduction

In the context of global malignancies, gastric cancer represents a significant health burden, ranking as the fifth most prevalent cancer and fourth leading cause of cancer‐related mortality worldwide [1, 2]. Despite therapeutic innovations, the challenge of poor 5‐year survival rates persists, complicated by delayed diagnoses and the incomplete understanding of the complex intracellular interactions of the tumour microenvironment [3, 4, 5, 6]. This complex ecosystem mediates sophisticated interplay among diverse cellular populations, from malignant cells to immune cells, tissue‐resident fibroblasts to interconnected endothelial cells, all engaged in complex molecular interactions.

The advance of single‐cell RNA sequencing technology has fundamentally improved our analytical capabilities [7, 8, 9]. This advanced approach has revealed previously uncharacterised cellular states and communication networks, offering unprecedented single‐cell resolution of the tumour microenvironment's heterogeneous composition. Within this complex interaction network, the distinct roles of cellular components in tumour modulation present a significant research question.

While extensive research has investigated tumour‐associated stromal elements, a critical knowledge gap still persists: The differential influences of normal adjacent tissue versus gastric mucosa on tumour progression remain poorly understood [10, 11]. This distinction carries significant implications, as normal adjacent tissue, despite its physical proximity to malignant zones, exhibits distinct molecular and cellular signatures compared to healthy gastric mucosa. The tissue‐specific senders of molecular signals variations potentially indicate undiscovered therapeutic opportunities.

Modern computational frameworks have improved our ability to analyse intracellular communication networks, enabling systematic mapping of ligand–receptor interactions within the tumour microenvironment [12, 13, 14]. However, comprehensive comparative analyses of cellular communication patterns between distinct tissue components and tumour cells in gastric cancer remain poorly understood. The endothelin axis, among various signalling cascades, has emerged as a significant pathway. Although implicated across multiple cancer types, its tissue‐specific role in gastric cancer communication networks requires deeper investigation. Using gastric cancer cell lines, we confirmed that the inhibition of EDNRA indeed downregulated cell proliferation and upregulated apoptosis. Additionally, TISIDB analysis revealed significant positive correlations between EDNRA and multiple immunostimulators, CXCL12, ENTPD1, TMEM173 and TNFSF4, suggesting the role of EDNRA in immunomodulation networks.

Our study utilises integrated single‐cell transcriptomics data from two independent researches to systematically characterise the intracellular communication patterns among normal adjacent tissue, gastric mucosa and tumour tissue. This systematic approach led to a significant finding: the previously uncharacterised role of endothelin receptor A (EDNRA) signalling in mediating gastric mucosa–tumour communication. These findings not only advance our understanding of the microenvironment within gastric cancer but also suggest promising therapeutic strategies for gastric cancer intervention.

## 2 Materials and Methods

### 2.1 Data Integration and Single‐Cell RNA Sequencing Analysis

Our study included an extensive analysis of transcriptomes from GSE183904 [7] and GSE184198 [15] cohorts. For the following analysis, we implemented a comprehensive computational pipeline combining integration, clustering and annotation. Within GSE183904, 48 surgical specimens represented 31 gastric cancer patients, including nine with matched tissue pairs. Within GSE184198, gastric cancer tissues and gastric mucosa tissues were integrated in the following analysis. We then merged two datasets containing 173,980 cells across three conditions (normal, tumour and GM tissues). Quality control followed strict criteria: cells failing to meet our established parameters—harbouring fewer than 200 genes, exceeding 6000 genes, or showing high mitochondrial content (> 10%) were excluded. Integration proceeded by Seurat v5 CCAIntegration method to correct batch effects while preserving biological variation. Following normalisation and variable feature identification, the dimensional landscape took shape via 30 principal components, while cellular identities were determined through unsupervised clustering. Specific markers—CD8A, IL2RA, STAT3, KLRD1, MS4A1, KIT, TNFRSF, CD163, PLD4, FN1, LUM, DCN, PLVAP, RGS5, NOTCH3, ACKR1, CDH1, EPCAM, MUC5AC, TFF1, REG4, TFF3, LIPF and PGA3—identified diverse cell populations. Integration quality was evaluated through mixing score calculations and systematic batch effect evaluations.

### 2.2 Cell–Cell Communication Analysis

To analyse complex cellular interactions, we utilised CellChat [12]. Expression data were first normalised and imported into CellChat with cellular metadata. The Human ligand–receptor interaction database (CellChatDB.human) was utilised in the following analysis. The computational workflow contained three key steps: (1) identifying over expressed genes and interactions, (2) commutating communication probabilities between cell populations, with interactions filtered to include only those from populations with at least 10 cells and (3) inferencing signalling pathways via computeCommunProbPathway function. This analysis identified distinct communication networks: 7557 interactions in normal‐to‐tumour communication versus 7320 in GM‐to‐tumour signalling. Significance was determined through statistical probability thresholds (*p* < 0.05). We quantified interaction strengths using the computeCommunProb function, while pathway analysis identified intracellular communication patterns. The comparison between normal‐to‐tumour and GM‐to‐tumour communications was performed through detailed differential interaction analysis. Circle plots and heatmaps visualised both specific interactions and broader communication patterns, with contribution analysis identifying key ligand–receptor pairs.

### 2.3 Survival Analysis

TCGA‐STAD data underwent comprehensive survival analysis through TCGAbiolinks [16, 17] and summarized experiment [18]. Raw expression data underwent careful preprocessing. GDCquery and GDCprepare transformed complex genomic information into analysable formats. When faced with duplicate samples, maximum expression values guided selection. The survminer package revealed optimal expression cut points through sophisticated algorithms, while Kaplan–Meier curves tracked patient outcomes across 100 months. Risk tables and confidence intervals provided statistical context, with distinct colour highlighting survival differences. Time intervals were divided into 20‐month segments for optimal visualisation.

### 2.4 Cell Culture and Reagents

In vitro studies centred on GSE‐1 epithelial cells and four gastric cancer lines: MKN‐45, NCI‐N87, MKN‐28 and HGC‐27. These cellular populations were cultured in RPMI‐1640 medium under precisely controlled conditions (37°C, 5% CO_2_). Monthly mycoplasma tests and biannual STR profiling were conducted to ensure culture authenticity. For molecular interventions, we prepared ENDRA selective antagonist ABT‐627 (2R‐(4‐methoxyphenyl)‐4S‐(1,3‐benzodioxol‐5‐yl)‐1‐(N,N‐di(n‐butyl)aminocarbonyl‐methyl)‐pyrrolidine‐3R‐carboxylic acid, MedChemExpress, HY‐15403) in DMSO (10 mM) [19] and endothelin‐1 (ThermoFisher, J66977.LB0) in water (100 μM), maintaining DMSO below 0.1% to preserve cellular integrity. Cell morphology and growth characteristics underwent daily monitoring, with fresh medium replenishment every 48 h. The working concentration of ABT‐627 was 50 μM based on previous studies [20]. Cultures maintained 70%–80% confluence throughout experimental procedures.

### 2.5 RNA Analysis

RNA isolation employed TRIzol methodology, yielding high‐purity transcripts (260/280: 1.8–2.0). Beyond spectrophotometric analysis, gel electrophoresis confirmed RNA integrity based on sharp 28S and 18S rRNA bands. The reverse transcription protocol utilised both oligo‐dT and random primers with the PrimeScript RT Kit, optimising cDNA synthesis from varied RNA populations. qPCR cycling conditions (95°C/15 s, 60°C/60 s for 40 cycles) followed initial denaturation (95°C, 10 min). Melting curves validated amplification specificity. GAPDH served as the normalisation standard. Each primer pair underwent efficiency validation through standard curve analysis.

### 2.6 Western Blot Analysis

The whole protein was extracted using RIPA buffer supplemented with protease and phosphatase inhibitor cocktails and then quantified using BCA assay. After transferring (200 mA, 2 h), membranes were blocked by the blocking solution (5% nonfat milk, 1 h, room temperature) followed by primary antibodies: EDNRA (Abcam, ab117521, 1:1000), EDNRB (ThermoFisher, PA3‐066, 1:1000), Bcl‐2 (Abcam, ab182858, 1:1000), Bax (Abcam, ab32503, 1:1000), cleaved caspases 3 (Cell signalling, 9661S, 1:1200), cleaved caspases 8 (Cell signalling, 9496S, 1:1000), cleaved caspases 9 (Cell signalling, 9509T, 1:1500), and GAPDH (ThermoFisher, MA5‐15738, 1:5000). After overnight incubation at 4°C, HRP‐conjugated secondary antibodies (1:3000) facilitated visualisation through enhanced chemiluminescence. Multiple exposure times ensured signal linearity.

### 2.7 Cell Proliferation Assay

We tracked cellular proliferation through CCK‐8 assays, seeding cells in 96‐well plates. After 12‐h attachment, cells encountered 50 μM of ABT‐627 or endothelin‐1 stimulation (100 nM). For overexpression studies, cells underwent Lipofectamine 3000‐mediated transfection following the manufacturer's optimised protocols. Empty vector controls validated specificity. Proliferation was measured at 0, 24, 48, 72, and 96 h through careful addition of CCK‐8 reagent (10 μL/well) and precise 2‐h incubation. Background correction used medium‐only wells, while edge wells contained PBS to minimise evaporation effects. Each experimental condition spanned five technical replicates, with three independent biological replicates ensuring reproducibility.

### 2.8 Statistical Analysis

Three independent replicates underpinned each experiment, with technical replicates ensuring measurement precision and reliability. R v4.0.3 facilitated data visualisation and statistical testing. Significance emerged through Student's *t*‐tests (paired comparisons) or ANOVA with Tukey's post hoc comparisons (multiple groups). Survival analyses employed log‐rank tests with hazard ratio calculations, while Pearson's coefficient quantified correlations. For quantification of western blotting results, statistical comparisons were performed using one‐way ANOVA followed by Tukey's honest significant difference post hoc test to account for multiple comparisons among different experiment groups. All statistical tests were two sided, and throughout all analyses, *p* < 0.05 marked statistical significance. Results are presented as mean ± standard deviation unless otherwise specified.

## 3 Results

### 3.1 Integrated Analysis Reveals Distinct Cell Populations in Gastric Cancer Microenvironment

**FIGURE 1:**
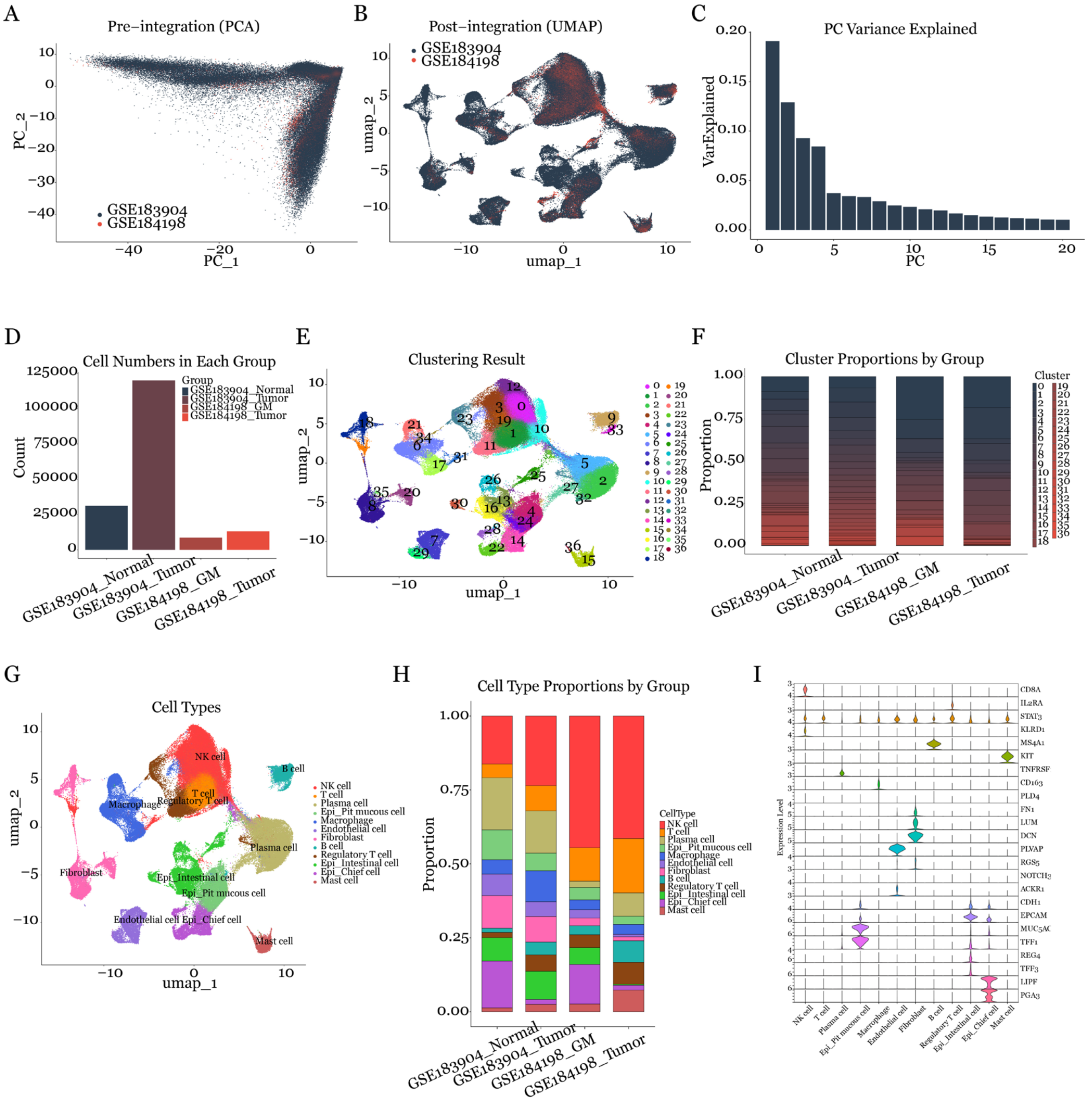
Integration analysis of single‐cell RNA sequencing data from gastric cancer datasets. (A) PCA plot revealing cell distribution before integration of GSE183904 and GSE184198 datasets, highlighting batch effects between sources. (B) UMAP visualisation of integrated data demonstrating successful batch effect correction through reciprocal PCA and harmony integration. (C) Bar plot illustrating variance explained by the first 20 principal components, with PC1 and PC2 accounting for major variation. (D) Distribution of cell numbers across groups: GSE183904_Normal (*n* = 31,198), GSE183904_Tumour (*n* = 119,878), GSE184198_GM (*n* = 8751) and GSE184198_Tumour (*n* = 13,153). (E) UMAP plot displaying 37 distinct clusters from unsupervised clustering. (F) Stacked bar plot showing normalised cluster proportions across groups. (G) UMAP visualisation of cell type annotations based on canonical markers, with each dot representing a single cell. (H) Cell type proportions among different groups, demonstrating tissue‐specific cellular composition. (I) Violin plot depicting expression levels and percentage of cells expressing canonical markers used for cell type annotation.

Our analysis combined a complex cellular landscape comprising 172,980 cells—151,076 from GSE183904 [7] dataset integrated with 21,904 from GSE184198 [15]. Initial PCA visualisation demonstrated batch effects between these two datasets (Figure 1A), yet post‐integration analysis aligned these populations, with cells clustered by biological identity rather than dataset origin (Figure 1B). The first twenty principal components demonstrated significant variation, with PC1 and PC2 alone explaining approximately 30% of total variance (Figure 1C). The distribution of cells across different conditions was quantified, with GSE183904_Normal (*n* = 31,198), GSE183904_Tumour (*n* = 119,878), GSE184198_GM (*n* = 8751), and GSE184198_Tumour (*n* = 13,153) (Figure 1D). In the integration step, dimensionality reduction through UMAP further showed the complex relationships among cell populations, while batch effect assessment confirmed successful data integration.

Unsupervised clustering identified 37 distinct populations (Figure 1E,F), which comprised 12 major cell types (Figure 1G). Cell composition analysis showed significant differences between tissue types, with tumour tissues showing notably increased proportions of immune cells, particularly NK cells and plasma cells, compared to normal and GM tissues (Figure 1H). Each type exhibited distinctive molecular signatures: NK cells expressed KLRD1, while T cells expressed CD8A. Plasma cells expressed TNFRSF, macrophages expressed CD163 and PLD4, and B cells expressed MS4A1. Regulatory T cells showed IL2RA and STAT3 expressions, while mast cells expressed KIT. The stromal components included fibroblasts expressing FN1, LUM and DCN, alongside PLVAP‐positive endothelial cells. Notably, epithelial populations consisted of three distinct subtypes: pit mucous cells (CDH1+/MUC5AC+/TFF1+), intestinal cells (CDH1+/REG4+/TFF3+) and chief cells (CDH1+/LIPF+/PGA3+) (Figure 1I).

### 3.2 Distinct Interaction Patterns in Normal‐To‐Tumour and GM‐To‐Tumour Communication

**FIGURE 2:**
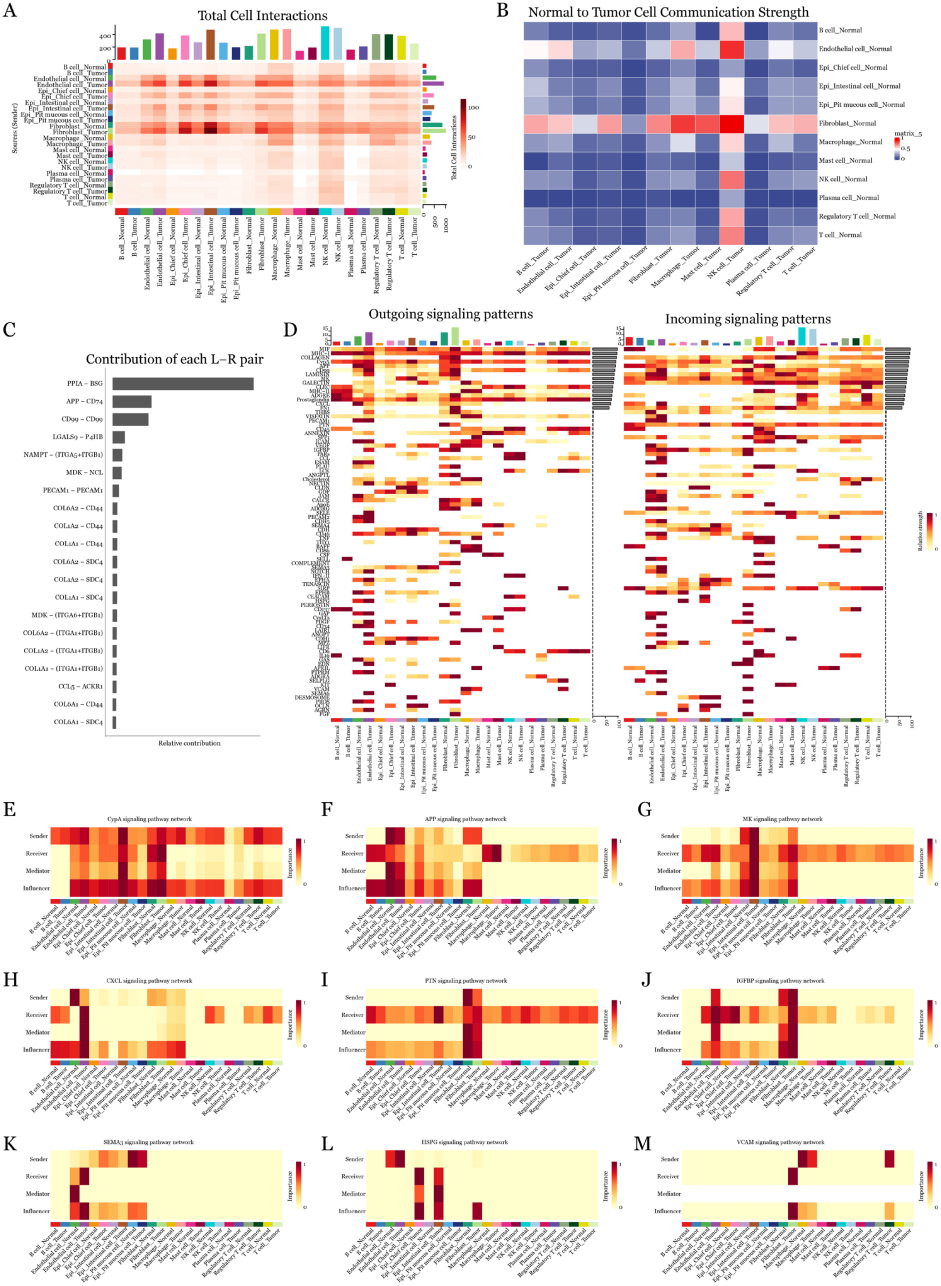
Analysis of normal‐to‐tumour cell communication. (A) Heatmap depicting total cell interactions between different cell types, with colour intensity reflecting interaction strength and scale representing normalised interaction counts. (B) Heatmap showing normalised cell communication strength from normal to tumour cells. (C) Ranked contribution of ligand–receptor pairs, weighted by expression levels. (D) Paired heatmaps illustrating outgoing and incoming signalling patterns across cell types, with separate normalisation for sending and receiving signals. (E–M) Network analysis heatmaps of major signalling pathways (CypA, APP, MK, CXCL, PTN, IGFBP, SEMA3, HSPG and VCAM), displaying sender, receiver, mediator and influencer.

Notable benchmarking research reveals CellChat's exceptional resilience against data perturbations and remarkable concordance with empirically confirmed cytokine activities, with the implementation of its sophisticated false‐positive filtering algorithms [21]. This methodological robustness in distinguishing authentic intercellular communication networks from spurious associations proved instrumental for our analysis. To identify the cellular interaction landscape between normal tissue and tumour cells, we performed a comprehensive CellChat analysis. The total interaction heatmap illuminated a complex network of cell–cell communications, with intensity gradients reflecting interaction strengths (Figure 2A). The normal‐to‐tumour communication strength heatmap unveiled preferential signalling patterns between specific cell types (Figure 2B), showing high interaction intensity between diverse cellular populations from both tumour and normal tissue. Detailed contribution analysis of ligand–receptor pairs highlighted the top 20 molecular ligand–receptor pairs, with PPIA‐BSG emerging as the predominant interaction (Figure 2C). The paired visualisation of outgoing and incoming signalling patterns revealed bidirectional communication dynamics (Figure 2D). Network analyses of nine major pathways demonstrated distinct roles—sender, receiver, mediator and influencer—for different cell populations in CypA, APP, MK, CXCL, PTN, IGFBP, SEMA3, HSPG and VCAM signalling networks (Figure 2E–M).

**FIGURE 3:**
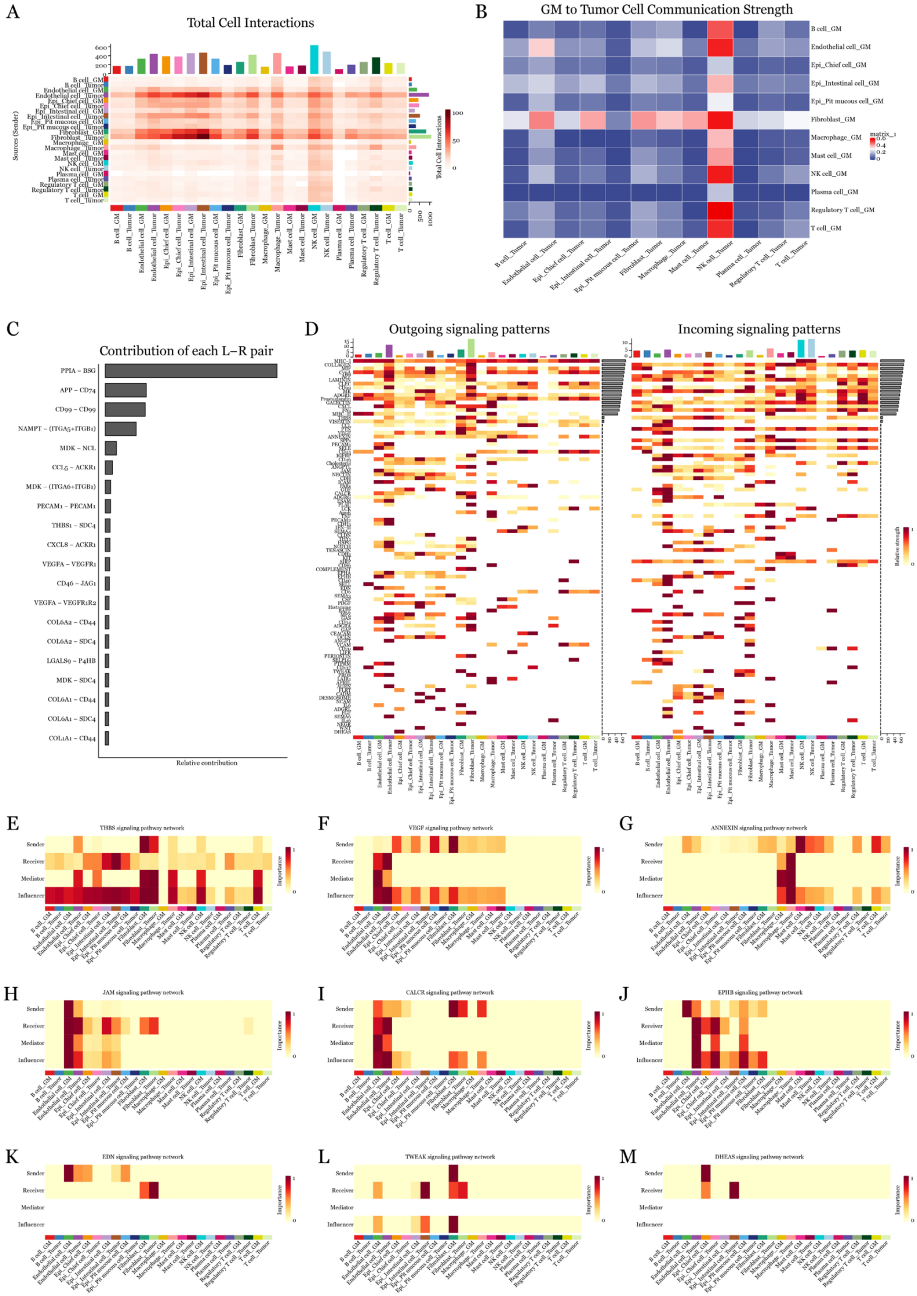
Analysis of GM‐to‐tumour cell communication. (A) Heatmap showing significant cell interactions between GM and tumour cells (*p* < 0.05). (B) Heatmap showing normalised cell communication strength from GM to tumour cells. (C) Bar plot showing relative contribution of each ligand–receptor pair in GM‐to‐tumour communication, filtered for significant interactions. (D) Paired heatmaps of outgoing and incoming signalling patterns, highlighting major communication axes. (E‐M) Network analysis of THBS, VEGF, ANNEXIN, JAM, CALCR, EPHB, EDN, TWEAK and DHEAS pathways, highlighting pathway‐specific patterns and key cellular players.

Parallel analysis of GM‐to‐tumour intracellular communications exposed tissue‐specific interaction patterns. The total interaction heatmap revealed distinct communication signatures compared to normal tissue (Figure 3A). GM‐to‐tumour communication strength analysis (Figure 3B) highlighted unique cellular partnerships. Contribution analysis identified tissue‐specific top 20 ligand–receptor pairs mediating these interactions (Figure 3C). The bidirectional signalling patterns showed remarkable differences from normal tissue communication (Figure 3D). Notably, pathway‐specific analyses uncovered differential activation of THBS, VEGF, ANNEXIN, JAM, CALCR, EPHB, EDN, TWEAK and DHEAS networks, suggesting tissue‐specific regulation of these signalling cascades (Figure 3E–M). These analyses collectively revealed fundamental differences in how GM and normal tissues communicate with tumour cells.

### 3.3 Differential Cell‐Cell Communication Patterns in Normal and GM Tissues

**FIGURE 4:**
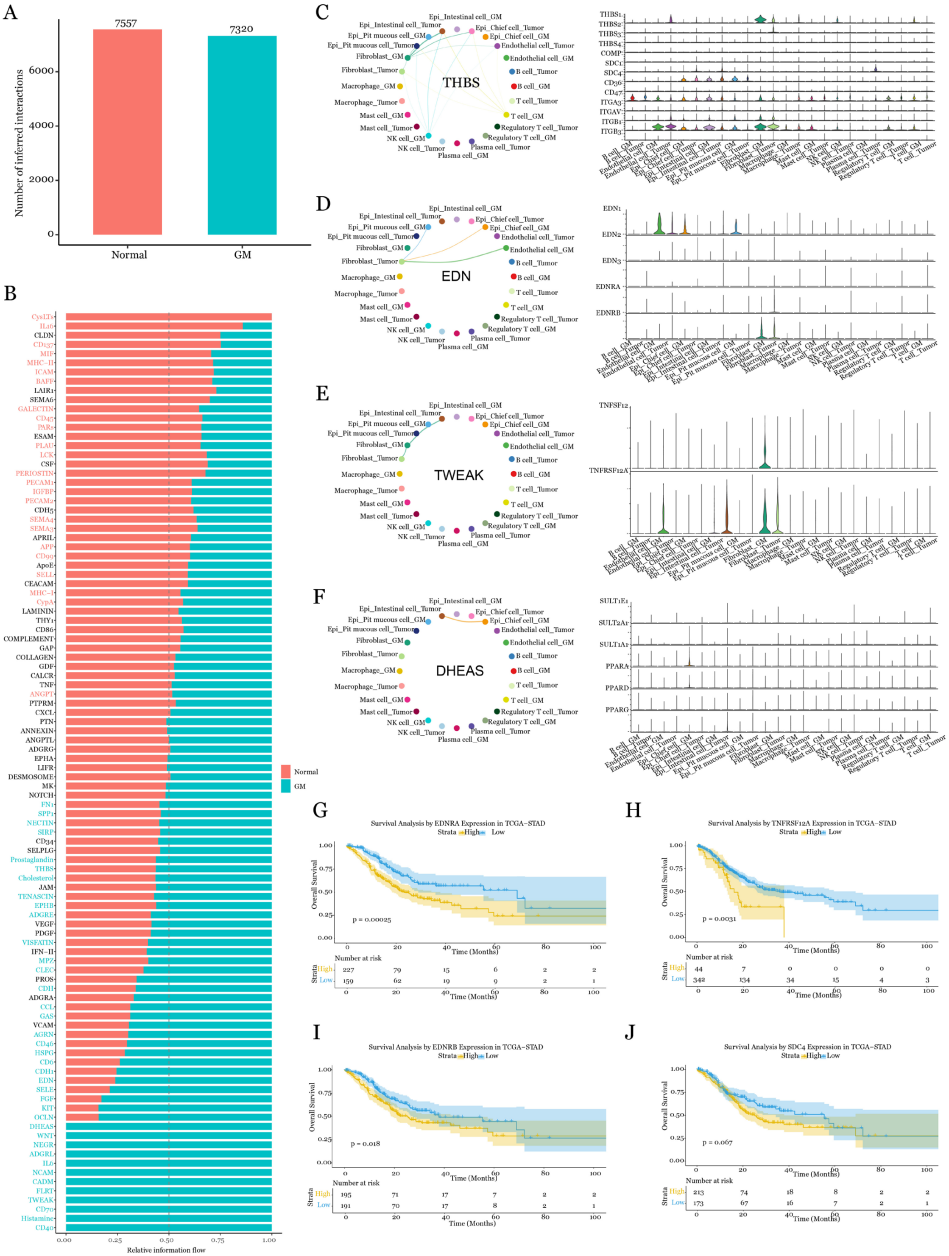
Identification of GM‐specific signalling pathways. (A) Comparison of interaction numbers between normal (*n* = 7557) and GM (*n* = 7320) communication with tumour cells, showing total significant interactions. (B) Pathway contribution barplot normalised by pathway size. (C‐F) Network visualisation and violin plots showing expression patterns of THBS, EDN, TWEAK, and DHEAS pathways across cell types, with network edges representing significant interactions (*p* < 0.05). (G‐J) Kaplan–Meier survival curves based on expression levels of EDNRA, EDNRB, EFNB2 and SDC4 in TCGA‐STAD dataset, with hazard ratios and confidence intervals shown.

CellChat analysis revealed significant differences in cellular interactions. Normal tissue exhibited 7557 significant interactions with tumour cells, while GM tissue showed 7320 distinct interactions (Figure 4A). Next, tissue‐specific patterns emerged, with certain cell populations showing enhanced communication in GM tissue. Quantitative analysis identified distinct interaction strengths between different cellular populations, demonstrating the complexity of tissue‐specific signalling networks (Figure 4B).

Detailed molecular mapping uncovered several pathways uniquely enriched in GM‐to‐tumour communication. The THBS signalling network revealed complex interactions between epithelial and stromal populations (Figure 4C), while EDN pathway analysis demonstrated strong activation in specific cellular populations (Figure 4D). TWEAK and DHEAS pathways showed distinctive expression patterns across different cell types, suggesting pathway‐specific regulation in the tumour microenvironment (Figure 4E,F). Most notably, survival analysis of TCGA‐STAD data revealed significant clinical implications. Among the identified pathways, high EDNRA expression strongly correlated with poor overall survival (*p* = 0.00025) (Figure 4G). Similar trends were observed for TNFRSF12A (*p* = 0.0031) (Figure 4H), EDNRB (*p* = 0.018) (Figure 4I) and SDC4 (*p* = 0.067) (Figure 4J). Each analysis included comprehensive risk tables showing the number of patients at risk over time, providing robust statistical support for the clinical significance of these pathways. These survival analyses collectively suggest that tissue‐specific signalling patterns have important prognostic implications in gastric cancer.

### 3.4 EDNRA Expression and Clinical Significance

**FIGURE 5:**
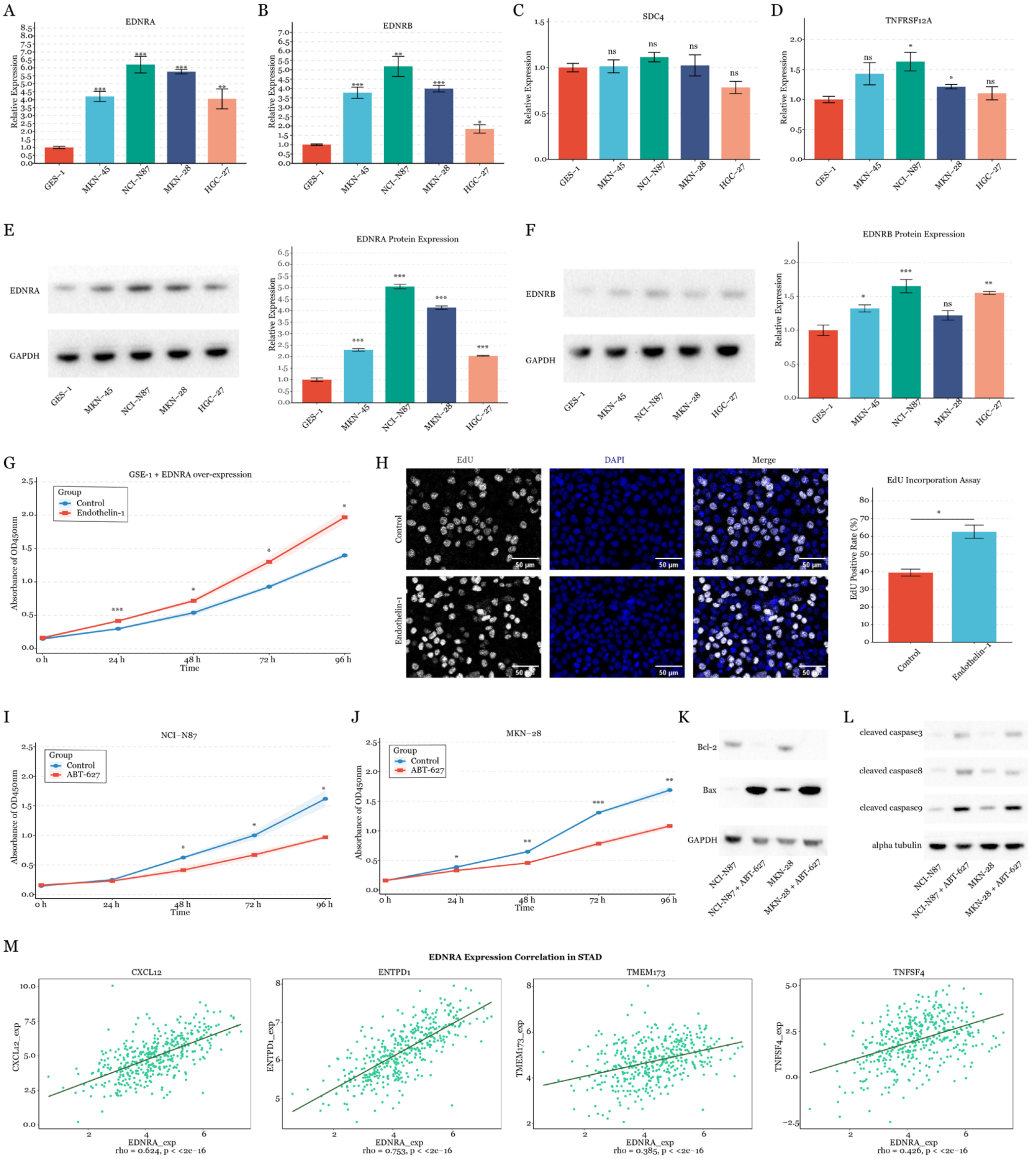
Functional validation of EDNRA signalling. (A‐D) qRT‐PCR analysis of EDNRA, EDNRB, SDC4 and TNFRSF12A expression in human gastric epithelial cell line (GSE‐1) and gastric cell lines (MKN‐45, NCI‐N87, MKN‐28 and HGC‐27). Data presented as mean ± SD from three independent experiments performed in triplicate. *p < 0.05, **p < 0.01, ***p < 0.001, ns: Not significant, determined by one‐way ANOVA with Tukey's post hoc test. (E‐F) Western blot analysis of EDNRA and EDNRB protein levels in indicated cell lines, with GAPDH as loading control. (G) Cell proliferation assay in GSE‐1 cells with EDNRA overexpression and endothelin‐1 (100 nM) treatment, measured at 0, 24, 48, 72 and 96 h using CCK‐8 assay. (H) EdU incorporation assay to measure the proliferation of in GSE‐1 cells with EDNRA overexpression and endothelin‐1 (100 nM) treatment. (I‐J) Cell proliferation assays in NCI‐N87 and MKN‐28 cells treated with ABT‐627 (50 μM) or control, showing time‐dependent growth inhibition. (K) Western blot analysis of Bcl‐2 and Bax expression in cells treated with ABT‐627 for 48 h, showing modulation of apoptotic regulators. (L) Western blot analysis of cleaved caspase‐3, −8 and − 9 levels in cells treated with ABT‐627 for 48 h, demonstrating activation of both intrinsic and extrinsic apoptotic pathways. (M) Correlation analysis of EDNRA expression with key immunostimulators in TCGA‐STAD dataset. All experiments were performed with three independent biological replicates (*n* = 3), with data presented as mean ± standard deviation.

Initial screening revealed distinct expression patterns of key signalling molecules across gastric cell lines. EDNRA showed marked upregulation in all gastric cancer cell lines examined (MKN‐45, NCI‐N87, MKN‐28 and HGC‐27) compared to normal gastric epithelial GSE‐1 cells, with the highest expression observed in NCI‐N87 and MKN‐28 (Figure 5A). Similarly, EDNRB expression exhibited cancer cell‐specific elevation, particularly in NCI‐N87 cells (Figure 5B). In contrast, SDC4 showed comparable expression levels across all cell lines (Figure 5C), while TNFRSF12A displayed moderate upregulation in NCI‐N87 cells (Figure 5D; *p < 0.05). To validate these transcriptional findings, we performed western blot analysis. EDNRA protein levels closely mirrored the mRNA expression patterns, confirming elevated expression in gastric cancer cell lines with particularly strong signals in NCI‐N87 and MKN‐28 cells (Figure 5E). EDNRB protein levels also increased in gastric cell lines (Figure 5F). Based on the expression results, we chose the most promising and significant EDNRA pathway to examine its biological function.

### 3.5 Functional Validation of EDNRA Signalling

Next, functional studies demonstrated EDNRA's significant impact on cellular behaviour. EDNRA overexpression in GSE‐1 cells significantly enhanced proliferation upon endothelin‐1 stimulation, showing a significant increase by 96 h (Figure 5G). Also, EdU incorporation assay results confirmed that EDNRA enhanced GSE‐1 cell proliferation with endothelin‐1 treatment (Figure 5H). ABT‐627 is a potent, orally bioavailable endothelin antagonist with high selectivity for the endothelin receptor A (Ki = 0.034 nm) over the endothelin receptor B (Ki = 63.3 nm), demonstrating 1800‐fold selectivity [22]. This compound effectively blocks the biological effects of ET‐1 by inhibiting mitogen and vascular biological responses induced by endothelin 1 challenge. Research has shown that the combination of ABT627 and Taxotere treatment caused greater antiproliferative and proapoptotic activity in vitro, as well as antitumour activity in vivo [20]. Similarly, the inhibition of EDNRA by ABT‐627 reduced growth in both NCI‐N87 and MKN‐28 gastric cancer cells significantly within 96 h (Figure 5I,J). Time‐course analysis showed the consistent nature of these effects, with significant differences observed as early as 48 h post‐treatment. These findings establish EDNRA as a key regulator of gastric cancer cell proliferation and survival.

### 3.6 Mechanistic Studies of EDNRA Inhibition

Additionally, molecular analysis showed ABT‐627's significant effects on the apoptotic machinery. Western blot demonstrated Bcl‐2 downregulation alongside Bax upregulation with treatment of ABT‐627 in both NCI‐N87 and MKN‐28 gastric cancer cells (Figure 5K). The compound activated both intrinsic and extrinsic apoptotic pathways—caspase‐8 activation indicated death receptor pathway engagement, while increased caspase‐9 cleavage suggested mitochondrial pathway involvement. Increased cleaved caspase‐3 levels also confirmed significant apoptotic activation (Figure 5L). This dual pathway activation explains ABT‐627's anti‐proliferative effects, suggesting its potential therapeutic application in gastric cancer treatment, particularly in cases with elevated EDNRA expression. We then conducted correlation analysis of EDNRA expression with key immunomodulatory molecules in the TCGA‐STAD dataset using TISIDB [23]. Results show significant positive correlations between EDNRA expression and the expression of multiple immunostimulators, including CXCL 12 (rho = 0.624), ENTPD1 (rho = 0.753), TMEM173 (rho = 0.385) and TNFSF4 (rho = 0.426) (Figure 5M). These strong correlations suggest that EDNRA signalling may interact with immunostimulatory pathways, potentially enhancing the tumour activation within the microenvironment in gastric cancer.

## 4 Discussion

### 4.1 Novel Insights Into Tissue‐Specific Communication in Gastric Cancer

Our integrated single‐cell analysis identifies previously uncharacterized communication patterns in the gastric cancer microenvironment [24, 25]. The identification of distinct interaction networks—7557 in normal‐to‐tumour versus 7320 in GM‐to‐tumour communication—gives us the power to identify tissue‐specific regulatory mechanisms. Significantly, the endothelin axis demonstrates specific activation in GM‐to‐tumour communication, with EDNRA identified as a key receptor. Analysis showed endothelial cells and epithelial cells from GM tissue predominantly express endothelin ligands, while tumour cells highly express EDNRA, suggesting a specific paracrine signalling mechanism. This spatial organisation provides new evidence regarding the role of non‐malignant tissue in cancer studies. The organisation of signalling components enhances our understanding of tissue‐context influences on tumour progression and may explain varying therapeutic responses across anatomical locations. These findings emphasise the importance of considering tissue‐specific effects in therapeutic development.

### 4.2 The Endothelin Axis as a Therapeutic Target

The endothelin receptor type A (EDNRA) belongs to the G protein‐coupled receptor family and plays crucial roles in multiple physiological processes including vasoconstriction, cell proliferation and tissue development [26, 27, 28, 29, 30, 31]. While its involvement has been documented in various cancers, its tissue‐specific role in gastric cancer progression remained largely unexplored. Our results on EDNRA overexpression and its correlation with poor survival indicate remarkable potential for developing selective EDNRA antagonists as potential target therapies for gastric cancer, especially for patients with confirmed high expression level of EDNRA, who might benefit from personalised treatments.

The functional validation of EDNRA signalling demonstrates its significant role in gastric cancer progression. ABT‐627, the specific inhibitor of EDNRA, shows significant efficacy by modulating the proliferation of cancer cells and activating both intrinsic and extrinsic apoptotic pathways. This dual mechanism is particularly relevant given EDNRA's correlation with poor survival in TCGA data. The compound's effects on Bcl‐2 downregulation and Bax upregulation, alongside caspase activation, suggest an effective anti‐cancer mechanism that may reduce resistance development [32, 33, 34, 35]. Furthermore, EDNRA's tissue‐specific activation pattern indicates its potential as both a prognostic marker and therapeutic target, suggesting opportunities for personalised treatment strategies. The correlation between EDNRA expression and clinical outcomes also supports its potential as a therapeutic target. The observed impact on cancer cell survival mechanisms, combined with tissue‐specific activation, indicates potential applications in stratified patient treatment approaches.

### 4.3 Clinical Implications and Future Directions

The tissue‐specific nature of endothelin signalling suggests the need for revised cancer therapy approaches. ABT‐627's anti‐tumour effects support further clinical trials, particularly in patients with high EDNRA expression [19, 36, 37, 38, 39]. Important research questions include: How does tissue‐specific EDNRA activation affect treatment responses? Can combination therapies targeting both EDNRA and other survival pathways produce synergistic effects? What biomarkers can predict response to EDNRA inhibition? Additional studies of tissue‐specific intracellular communication patterns in other cancer types may broaden therapeutic applications and improve the effects. The development of endothelin axis‐based biomarkers may improve personalised treatment strategies, while understanding the downstream mechanisms of the tissue‐specific signalling patterns may enhance cancer therapy. Future studies should investigate potential resistance mechanisms and optimal drug combinations to improve therapeutic efficacy [40, 41, 42]. EDNRA signalling interacts with multiple downstream effectors, potentially offering stronger therapeutic targets for gastric cancer treatment. Thoroughly understanding the crosstalk between EDNRA signalling and other pathways could reveal opportunities for synergistic inhibition with enhanced an‐tumour effect and potentially therapeutic effects. Combined targeting the network of EDNRA signalling might achieve more effective cancer treatment than monotherapy approaches.

## 5 Limitations

Our results provide valuable insights into EDNRA's role in gastric cancer progression but are limited in capturing how EDN signalling evolves over time during gastric cancer progression. While we have shown significant effects of EDNRA modulating cancer cell behaviours, we cannot track the dynamic changes of the signalling network. Future longitudinal studies with serial sampling would better understand the temporal dynamics of EDN signalling during the progression of gastric cancer. Also, cell lines offer reproducible experimental conditions for molecular mechanisms in our study; however, they may not fully recapitulate the complex heterogeneity and interactions in primary gastric tumours. Despite using multiple gastric cancer cell lines to validate our findings, these models still lack the tissue architecture that influences EDN signalling in vivo. Future validations using patient‐derived organoids would provide valuable and more physiologically relevant conditions to confirm the clinical potential of the EDN signalling.

## Author Contributions

**Xiaobin Zhu:** conceptualization (lead), data curation (lead), formal analysis (lead), investigation (lead), methodology (lead), project administration (lead). **Yating Zhang:** data curation (lead), formal analysis (lead), investigation (lead), methodology (lead), project administration (lead). **Hanlin Liao:** data curation (supporting), formal analysis (supporting), investigation (supporting), methodology (supporting). **Jing Hu:** data curation (supporting), formal analysis (supporting), investigation (supporting), methodology (supporting). **Xiao Xiao:** conceptualization (lead), resources (lead), supervision (lead), writing – original draft (lead), writing – review and editing (lead).

## Conflicts of Interest

The authors declare no conflicts of interest.

## Data Availability

All data analyzed in this study are fully presented within the manuscript in the form of tables and figures. No additional Supporting Information or external data repositories were used.
